# Genome-wide investigation and transcriptional analysis of cytosine-5 DNA methyltransferase and DNA demethylase gene families in tea plant (*Camellia sinensis*) under abiotic stress and withering processing

**DOI:** 10.7717/peerj.8432

**Published:** 2020-01-14

**Authors:** Chen Zhu, Shuting Zhang, Chengzhe Zhou, Lan Chen, Haifeng Fu, Xiaozhen Li, Yuling Lin, Zhongxiong Lai, Yuqiong Guo

**Affiliations:** 1 College of Horticulture, Fujian Agriculture and Forestry University, Fuzhou, Fujian, China; 2 Institute of Horticultural Biotechnology, Fujian Agriculture and Forestry University, Fuzhou, Fujian, China

**Keywords:** *Camellia sinensis*, C5-MTase, dMTase, Stress response, Withering processing, Transcript abundance

## Abstract

DNA methylation is a highly conserved epigenetic modification involved in many biological processes, including growth and development, stress response, and secondary metabolism. In the plant kingdom, *cytosine*-*5 DNA methyltransferase* (*C5-MTase*) and *DNA demethylase* (*dMTase*) genes have been identified in some plant species. However, to the best of our knowledge, no investigator has focused on the identification and analysis of *C5-MTase* and *dMTase* genes in tea plants (*Camellia sinensis*) based on genome-wide levels. In this study, eight *CsC5-MTases* and four *dMTases* were identified in tea plants. These *CsC5-MTase* genes were divided into four subfamilies, including *CsMET*, *CsCMT*, *CsDRM* and *CsDNMT2*. The *CsdMTase* genes can be classified into *CsROS*, *CsDME* and *CsDML*. Based on conserved domain analysis of these genes, the gene loss and duplication events occurred during the evolution of *CsC5-MTase* and *CsdMTase*. Furthermore, multiple *cis*-acting elements were observed in the *CsC5-MTase* and *CsdMTase*, including light responsiveness, phytohormone responsiveness, stress responsiveness, and plant growth and development-related elements. Then, we investigated the transcript abundance of *CsC5-MTase* and *CsdMTase* under abiotic stress (cold and drought) and withering processing (white tea and oolong tea). Notably, most *CsC5-MTases*, except for *CsCMT1* and *CsCMT2*, were significantly downregulated under abiotic stress, while the transcript abundance of all four *CsdMTase* genes was significantly induced. Similarly, the same transcript abundance of *CsC5-MTase* and *CsdMTase* was found during withering processing of white tea and oolong tea, respectively. In total, our findings will provide a basis for the roles of *CsC5-MTase* and *CsdMTase* in response to abiotic stress and the potential functions of these two gene families in affecting tea flavor during tea withering processing.

## Introduction

Epigenetics is the mechanism by which gene expression and phenotype are changed without alterations in DNA sequence, and these changes can be passed on continuously with cell mitosis and meiosis. DNA methylation is not only the most common epigenetic modification but also is an important link between phenotype and genotype. Increasingly abundant evidence demonstrates that DNA methylation can be divided into three types, namely, 5-methylcytosine (5mC), N6-methyladenine and N4-methylcytosine (Chen, Zhao & He, 2016; Ratel et al., 2006; Zhang et al., 2015). In the plant kingdom, 5mC is the predominant form of DNA methylation, which transfers the methyl group from S-adenosyl methionine to the carbon-5 (C-5) atom in the pyrimidine ring of cytosine residues. Unlike in animals, 5mC methylation in plants usually occurs in the CG, CHG and CHH contexts (H represents A, C or T), where the methylation status of the CG context is most stable (Cokus et al., 2008). It has been reported that DNA methylation also plays a crucial role in genomic imprinting, X chromosome inactivation, transposon suppression, and gene silencing (Chan, Henderson & Jacobsen, 2005; Law & Jacobsen, 2010).

In plants, DNA methylation can be divided into two types: DNA maintenance methylation and de novo DNA methylation. Among these types, DNA methylation is mainly regulated by cytosine-5 DNA methyltransferase (C5-MTase), including methyltransferase (MET), chromomethylase (CMT), domains rearranged methylase (DRM), and de novo DNA methyltransferase 2 (DNMT2). The MET gene family was first originally isolated from *Arabidopsis thaliana*, including *AtMET1*, *AtMET2a*, *AtMET2b* and *AtMET3* (Genger et al., 1999). In the plant kingdom, *MET* is a major regulatory gene affecting the DNA methylation status of plants and plays a major role in maintaining methylation in the symmetric CG context. CMT is a plant-specific DNA methyltransferase whose main function is to maintain methylation in CHG contexts through a reinforcing loop between CHG methylation and histone H3 lysine 9 (H3K9) methylation (Du et al., 2012, 2014). In addition, previous research has shown that CMT2 can participate in the maintenance of CHH methylation in plants (Zemach et al., 2013). A total of three *CMT* genes from *A. thaliana* were identified by genome sequencing, namely, *AtCMT1*, *AtCMT2* and *AtCMT3*. In *CMT* mutants of *A. thaliana*, both CHG and CHH methylation decreased sharply (Stroud et al., 2013; Zemach et al., 2013). Similarly, it has been reported that the absence of *CMT2* in *Zea mays* also results in the depletion of CHG methylation (Papa et al., 2001). Moreover, previous research has shown that DNMT2 also has C5-MTase activity (Tang et al., 2003). *DNMT2* is the smallest MET gene family found in different eukaryotes. Due to the considerable variability in the specific target recognition domain, DNMT2 is also involved in RNA methylation modification (Jeltsch et al., 2017; Vieira et al., 2017). DRM is homologous to DNMT3 in mammals, and both differ only in the order of related catalytic domains (Cao et al., 2000). In plants, DRM is primarily responsible for catalyzing de novo DNA methylation in all known DNA sequence contexts (CG, CHG and CHH) through the RNA-directed DNA methylation pathway. DRM2 also plays a vital regulatory role in maintaining methylation in the asymmetric CHH context (Gent et al., 2013). After the targeted destruction of the *DRM2* gene in rice, it was found that the genomic DNA methylation level was abnormal and adversely affected rice growth (Moritoh et al., 2012). In addition, double mutants (*drm1–drm2*) block de novo DNA methylation, resulting in a significant reduction in CHG methylation (Cao & Jacobsen, 2002).

The level of DNA methylation in plants is dynamic and depends not only on the establishment and maintenance of DNA methylation but also on DNA demethylation. DNA demethylation can be divided into passive demethylation and active demethylation. After DNA replication, the newly synthesized DNA strand cannot be methylated due to the maintenance of DNA methyltransferase inactivation, which is called passive methylation. Active demethylation is not dependent on DNA replication and is caused by a series of DNA demethylase (dMTase) catalysis. Therefore, the overall level of genomic DNA methylation is affected by both DNA methylase and demethylase. In contrast to DNA methylation catalyzed by a single *C5-MTase* gene, a series of genes are required for DNA demethylation, including *repressor of silencing 1* (*ROS1*), *demeter* (*DME*), *demeter-like 2* (*DML2*) and *demeter-like 3* (*DML3*) (Gong et al., 2002; Ortega-Galisteo et al., 2008). On the basis of the base excision repair mechanism, these dMTases recognize and excise the 5mC base and replace it with an unmethylated cytosine. In the *ros1* mutant of *A. thaliana*, both CHH and CHG methylation increased in the transposon region, and many genes were found to be highly methylated and silenced at the transcriptional level (Wang et al., 2016a; Yamamuro et al., 2014). Moreover, the expression level of some genes in the triple mutant (*ros1–dml2–dml3*) of *A*. *thaliana* was suppressed due to hypermethylation of the upstream promoter regions (Zhang, Lang & Zhu, 2018). Additionally, previous studies have shown that DME was more associated with genomic demethylation during pollen development (Schoft et al., 2011; Zhu, 2009). Deletion of these four *dMTase* genes leads to increased genomic methylation levels and hypermethylation of locus-specific DNA (Penterman et al., 2007).

Extensive studies have shown that dynamic changes in plant methylation levels are importantly associated with various abiotic stresses, including drought stress (Liu et al., 2018a; Sanchez & Paszkowski, 2014), cold stress (Steward et al., 2002), salt stress (Su et al., 2018; Sun et al., 2018), and ultraviolet (UV)/light radiation stresses (Eichten & Springer, 2015; Ganguly et al., 2018). In addition, DNA methylation also plays an important role in regulating growth and development (Liu et al., 2015; Xing et al., 2015) and secondary metabolism (Bharti et al., 2015; Zhang et al., 2016). Since DNA methylation is involved in the regulation of a wide range of biological processes, *C5-MTase* and *dMTase* genes have been extensively identified and analyzed in several plant species, including *A. thaliana* (Ogneva, Dubrovina & Kiselev, 2016), *Solanum lycopersicum* (Cao et al., 2014), *Arachis hypogaea* (Wang et al., 2016b), *Cynara cardunculus* (Gianoglio et al., 2017) and *Ricinus communis* (Victoria et al., 2018). However, to the best of our knowledge, no investigator focused on the identification and analysis of *C5-MTase* and *dMTase* genes in tea plants based on genome-wide levels.

Tea plant (*Camellia sinensis*) is an important economic crop that originates in China. With the extreme global climate, cold stress and drought stress may adversely affect the growth and development of tea plants and the quality of tea products. Previous studies have shown that plant methylation plays a crucial role in response to abiotic stress (Zhang, Lang & Zhu, 2018). Additionally, a recent report revealed that methylation levels are affected by *C5-MTase* and *dMTase* genes, and DNA methylation regulating transposon silencing may play a crucial role in genome size expansion (Wang et al., 2019a). However, no research has been published on exploring the roles of *C5-MTase* and *dMTase* genes in tea plants under abiotic stress. Furthermore, withering is the first indispensable process for improving flavors in the postharvest processing of oolong tea and white tea, which is closely associated with their unique aromas and flavors. Similar to tea plants are affected by stress, and fresh leaves (FL) are also affected by various stresses during different types of withering. During solar-withering, tea leaves may suffer from different types of stresses, including drought, heat, and UV/light radiation. However, the tea leaves are primarily affected by drought stress during indoor withering. The functions of *C5-MTase* and *dMTase* genes in the tea withering process have not been determined. With the release of the tea reference genome (Wei et al., 2018; Xia et al., 2017), it is possible to accurately identify *C5-MTase* and *dMTase* genes in tea plants. In the present study, all members of *C5-MTase* and *dMTase* were identified in tea plants, and their physical and chemical characteristics, phylogenetic relationships, gene structures, protein–protein interaction, and *cis-*acting elements were investigated. Moreover, the transcript abundance of *C5-MTase* and *dMTase* genes under various stresses and withering processing were analyzed. Our analysis provides valuable information for screening the *C5-MTase* and *dMTase* genes and their transcript abundance under abiotic stress and withering processing in tea plants. The results of this study may help to further elucidate the functional roles of the *C5-MTase* and *dMTase* genes in tea plants.

## Materials and Methods

### Genome-wide identification of *C5-MTase* and *dMTase* genes in tea plants

To perform genome-wide identification of *C5-MTase* and *dMTase* genes in tea plants, the known C5-MTase and dMTase sequences in *A. thaliana* (Table S1) were downloaded from The Arabidopsis Information Resource database (https://www.arabidopsis.org). These sequences were used to search against the tea reference genome (Xia et al., 2017) using the BLAST algorithm (*p*-value < 1.0E^−5^). Then, we obtained the putative C5-MTase and dMTase sequences in tea plants. To further verify the accuracy of these sequences in tea plants, we also downloaded the relevant sequences of C5-MTase and dMTase in *S. lycopersicum* (Table S1) from the Sol Genomics Network database (https://solgenomics.net). We also used these sequences in *S. lycopersicum* to search against the tea reference genome. The ProtParam tool (https://www.expasy.org/) and WoLF PSORT server (https://wolfpsort.hgc.jp/) were used to analyze the physical and chemical characteristics and the subcellular localizations of CsC5-MTase and CsdMTase, respectively. The percentage identity matrix of CsC5-MTase and CsdMTase was analyzed using DNAMAN 7.0 software. Moreover, the domain contained in CsC5-MTase and CsdMTase was identified and analyzed using the PfamScan tool (https://www.ebi.ac.uk/Tools/pfa/pfamscan/).

### Analysis of phylogenetic trees, gene structures, protein–protein interaction, and *cis*-acting elements

To further understand the classification of *C5-MTase* and *dMTase* genes, related C5-MTase and dMTase sequences from eight plants (*Oryza sativa*, *Glycine max*, *Z. mays*, *C. sinensis*, *A. thaliana*, *R. communis*, *C. cardunculus* and *S. lycopersicum*) were used to construct the phylogenetic tree using MEGA X software (Kumar et al., 2018) by the neighbor-joining method (bootstrap value = 1,000).

Based on genetic feature format data from the tea genome dataset, the gene structures of *C5-MTase* and *dMTase* in *C. sinensis* were analyzed using the Tbtools software (Chen et al., 2018). The motifs of C5-MTase and dMTase in *C. sinensis* were analyzed with MEME suite (Bailey et al., 2009), the maximum number of motifs was set as 10, and the remaining parameters were the default. The STRING 11 tool (https://string-db.org) was used to construct the protein–protein interaction network.

To analyze the *cis*-acting elements of *C5-MTase* and *dMTase*, the upstream sequences (2,000 bp) of the start codon were retrieved from the tea genome dataset, and then the *cis*-acting elements were analyzed using the PlantCARE tool (Lescot et al., 2002).

### Tea plant materials and treatments

The tea cultivar “Tieguanyin” was cultivated at Fujian Agriculture and Forestry University, Fuzhou, Fujian Province, China (E 119°14′, N 26°05′). To detect the transcript abundance of *CsC5-MTase* and *CsdMTase* in tea plants under cold stress, tea plants were subjected to cold temperature (4 °C). For drought treatments, tea plants were irrigated with 15% (w/v) PEG 4000 to simulate drought stress (Zhou et al., 2019). Then, the treated tea leaves were sampled at 0, 12, 24, 36 and 48 h.

For withering treatment of oolong tea, the fresh shoot and first to third leaves were uniformly picked from each tea plant. Then, the tea leaves were equally divided into three parts, each weighing 2 kg. The first part was collected without any processing. The second part was subjected to solar withering under sunlight for 45 min (temperature 25 ± 2 °C, relative humidity 60 ± 5%, illumination intensity 40,000 ± 1,000 Lx, and leaf layered thickness 1 cm). The third part of tea leaves were evenly layered and exposed to indoor light for 45 min (illumination intensity 100 ± 5 Lx). The other parameters in the indoor-withering processing were all consistent with those in the solar-withering processing. The FL, indoor-withered leaves (IW), and solar-withered leaves (SW) were collected for analyses in this study. The withering processing of white tea was performed according to the previous method (Yue et al., 2019) with minor modifications. For the withering treatment of white tea, one bud and two leaves were uniformly picked from each tea plant. The leaves were left in a withering room. The withered leaves of white tea were collected at 0, 12, 24, 36 and 48 h after withering.

All samples were frozen in liquid nitrogen immediately and stored at −80 °C for further experiments. Each sample was performed in three independent biological replicates.

### Total RNA extraction and transcript abundance analyses of *C5-MTase* and *dMTase* genes in tea plants under abiotic stress and withering processing

Total RNA was extracted using TransZol Up Reagent (TransGen Biotech, Beijing, China). The RNA integrity was checked by gel electrophoresis and micro-ultraviolet spectrophotometry (Nanodrop). Then, total RNA was reverse transcribed into first-strand cDNA quantitative real-time polymerase chain reaction (qRT-PCR) using a Transcript First-Strand cDNA Synthesis SuperMix (TransGen Biotech, Beijing, China). The qRT-PCR was performed using the LightCycler 480 platform (Roche Applied Sciences, Basel, Switzerland) with TransStart Tip Green qPCR SuperMix (TransGen Biotech, Beijing, China). The qRT-PCR procedure and reaction system were both used in the previous method (Guo et al., 2019). The *glyceraldehyde-3-phosphate dehydrogenase* and *β-actin* genes were used as reference genes. The transcript abundance was calculated using the 2^−ΔΔCt^ method (Livak & Schmittgen, 2001), and all primers used for qRT-PCR were designed using the Tea Plant Information Archive platform (Xia et al., 2019) (Table S2). All qRT-PCR analyses were performed in three biological replications, respectively. Statistical analyses were conducted using SPSS 25 software, and the data were analyzed by one-way analysis of variance followed by Tukey’s post-hoc test.

## Results

### Genome-wide identification and sequence feature analysis of *CsC5-MTase* and *CsdMTase* genes

**Figure 1:**
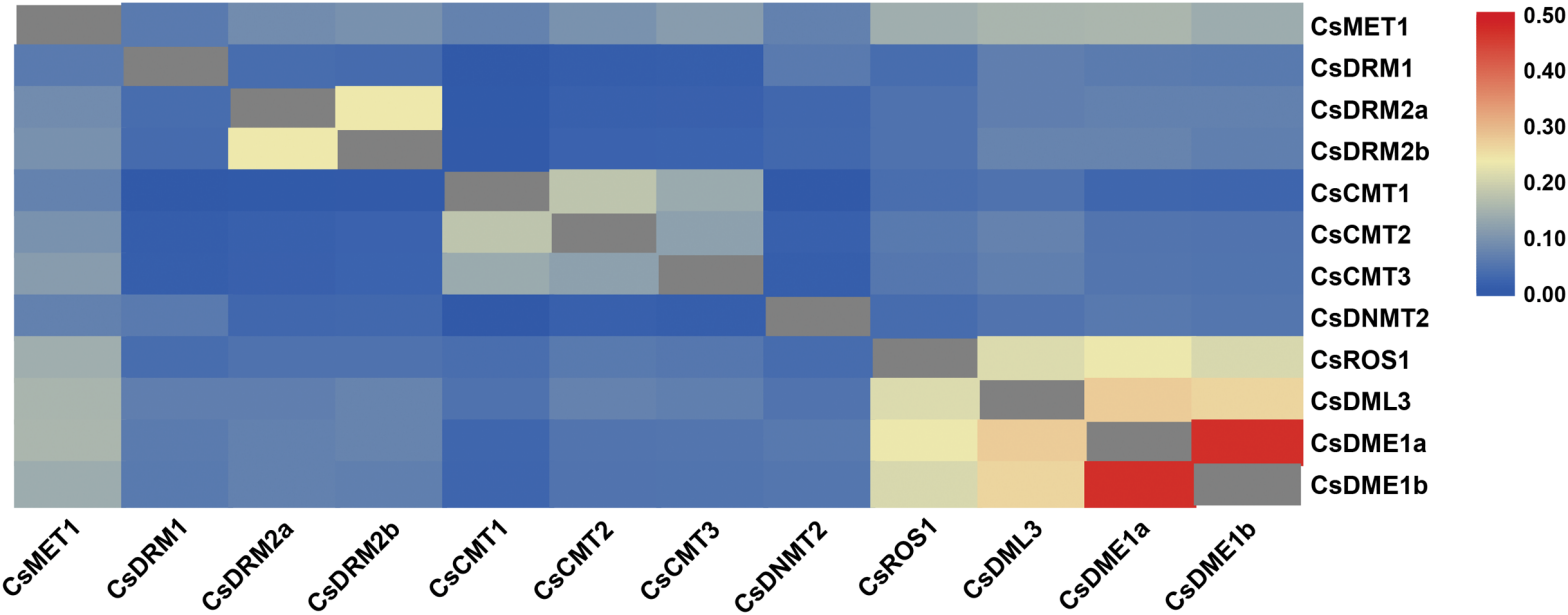
Sequence similarity of C5-MTases and dMTases in tea plant. The colored bar indicates the correlation of two proteins. Blue represents a low correlation; red represents a high correlation; gray represents no correlation analysis on these proteins.

After searching the tea reference genome, a total of eight *CsC5-MTase* and four *CsdMTase* genes were identified in tea plants. Among these genes, *CsC5-MTase* included one *CsMET* gene, three *CsDRM* genes, three *CsCMT* genes, and one *CsDNMT2* gene, while *CsdMTase* included one *CsROS* gene, one *CsDML* gene, and two *CsDME* genes. The sequence characteristic analysis showed that the open reading frames of eight *CsC5-MTase* genes ranged in size from 1,155 to 4,707 bp, while the ORF range of four *CsdMTase* genes was 3,015 to 5,856 bp (Table 1). The deduced proteins of CsC5-MTase varied between 384 and 1,568 amino acids. Among these proteins, the CsC5-MTase protein with the least number of amino acids is CsDNMT2, while CsMET1 contains the most amino acids. In CsdMTase proteins, protein length varied between 1,004 and 1,951 amino acids. The molecular weights of these proteins ranged from 42.99 to 217.83 kDa, and the theoretical *p*I values ranged from 5.03 to 8.19. Except for CsCMT1 and CsDME1a, the theoretical *p*I values of most CsC5-MTase and CsdMTase proteins were smaller than 7.0, indicating that these proteins were acidic. The instability index of CsDRM1, CsCMT1 and CsCMT3 were all smaller than 40, showing that these three proteins belong to stable protein, while the other nine proteins were unstable. According to grand average of hydropathicity (GRAVY) analysis, all CsC5-MTase and CsdMTase proteins were hydrophilic. In addition, the aliphatic index of these proteins ranged from 61.61 to 86.84. According to the result of putative subcellular localization, only CsDRM2b is located in the cytoplasm, whereas the other 11 proteins are localized in the nucleus. This result was consistent with previous findings that different RcDRM members are located in multiple organelles in *R. communis* (Victoria et al., 2018). The sequence similarity of these CsC5-MTase and CsdMTase proteins was also analyzed (Fig. 1). Compared with dMTase proteins, all C5-MTase proteins shared a low level of identity with each other, indicating that different members of C5-MTase proteins contain diverse functions. The sequences of CsDRM2a and CsDRM2b have significantly higher similarities than other CsC5-MTase proteins. In CsdMTase, CsDME1a and CsDME1b have the highest similarity. These results indicated that there are some duplicated genes in the *CsC5-MTase* and *CsdMTase* genes.

**Table 1:** Summary information of *CsC5-MTase* and *CsdMTase* genes in tea plant.

Gene name	Genome ID	ORF (bp)	Amino acid (aa)	Molecular weight (kDa)	Theoretical *p*I	Instability index	Aliphatic index	GRAVY	Subcellular localization
CsMET1	CSA018787.1	4,707	1,568	176.58	5.83	44.71	73.62	−0.522	Nucleus
CsDRM1	CSA001898.1	1,410	469	52.98	7.17	39.64	86.84	−0.325	Nucleus
CsDRM2a	CSA001531.1	1,863	620	69.16	5.03	41.31	78.15	−0.523	Nucleus
CsDRM2b	CSA005657.1	1,875	624	70.69	5.12	43.38	80.96	−0.382	Cytoplasm
CsCMT1	CSA032127.1	1,884	627	70.31	8.19	33.31	83.76	−0.293	Nucleus
CsCMT2	CSA030966.1	2,511	836	93.66	5.77	45.56	80.45	−0.384	Nucleus
CsCMT3	CSA021917.1	2,160	719	80.64	6.56	30.94	84.02	−0.263	Nucleus
CsDNMT2	CSA000025.1	1,155	384	42.99	5.19	53.88	70.31	−0.399	Nucleus
CsROS1	CSA023990.1	3,015	1,004	112.41	5.65	48.7	77.62	−0.529	Nucleus
CsDML3	CSA015244.1	5,856	1,951	217.83	6.68	43.95	70.11	−0.609	Nucleus
CsDME1a	CSA015772.1	5,718	1,905	212.99	8.04	48.83	63.8	−0.795	Nucleus
CsDME1b	CSA017793.1	5,691	1,896	211.51	6.85	47.07	61.61	−0.795	Nucleus

**Figure 2:**
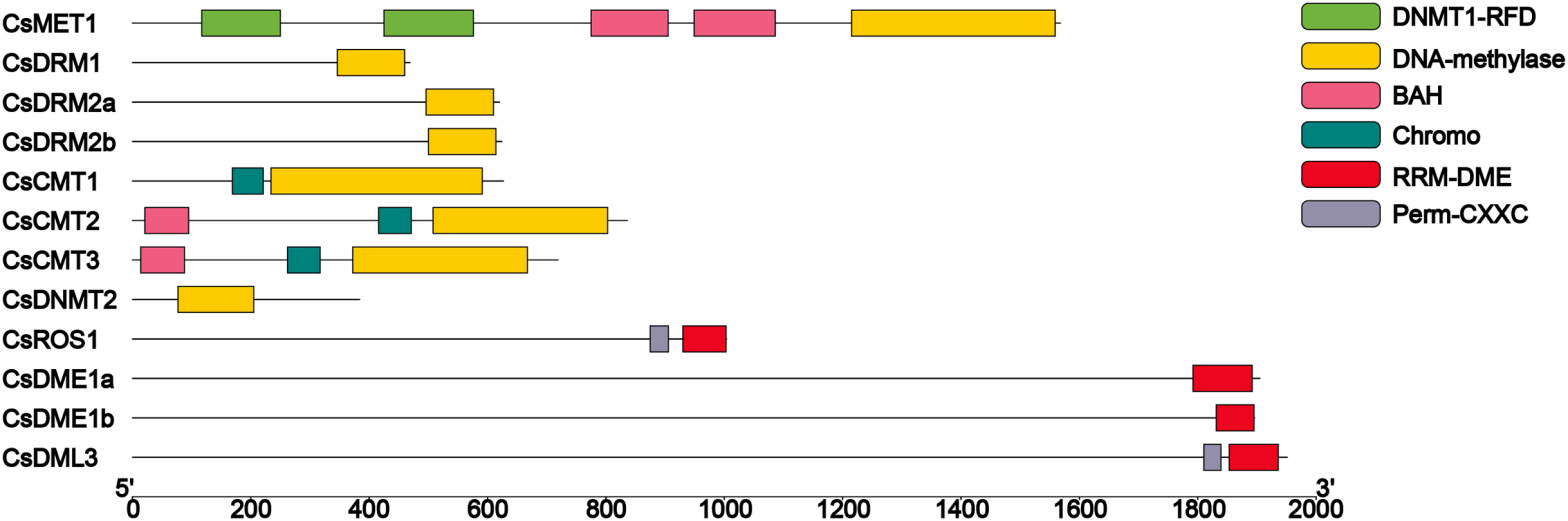
Conserved domain analysis of CsC5-MTases and CsdMTases.

On the basis of structural domain analysis (Fig. 2), all of the C5-MTase included the DNA-methylase domain (PF00145). According to whether there are other domains and the location of the DNA-methylase domain in sequences, eight CsC5-MTases can be divided into four subfamilies, namely, MET, DRM, CMT and DNMT2. The CsMET harbors two DNMT1-RFD domains (PF12047), two BAH domains (PF01426) and one DNA-methylase domain (PF00145). Compared to CsCMTs, three CsDRMs and one CsDNMT2 each contained a single DNA-methylase domain (PF00145). All three CsMETs include one Chromo domain (PF00385) and one DNA-methylase domain (PF00145). Unlike CsCMT1, both CsCMT2 and CsCMT3 have a BAH domain (PF01426). Domain analysis of CsdMTase revealed that all four subfamilies of CsdMTase contain an RRM-DME domain (PF15628) at their C-terminus. Except for CsDME, CsDML3 and CsROS1 contain an extra Perm-CXXC domain. These results indicated that CsROS and CsDML are more structurally similar to each other than to CsDME.

### Phylogenetic classification, structures and motif analysis of *CsC5-MTase* and *CsdMTase* genes

**Figure 3:**
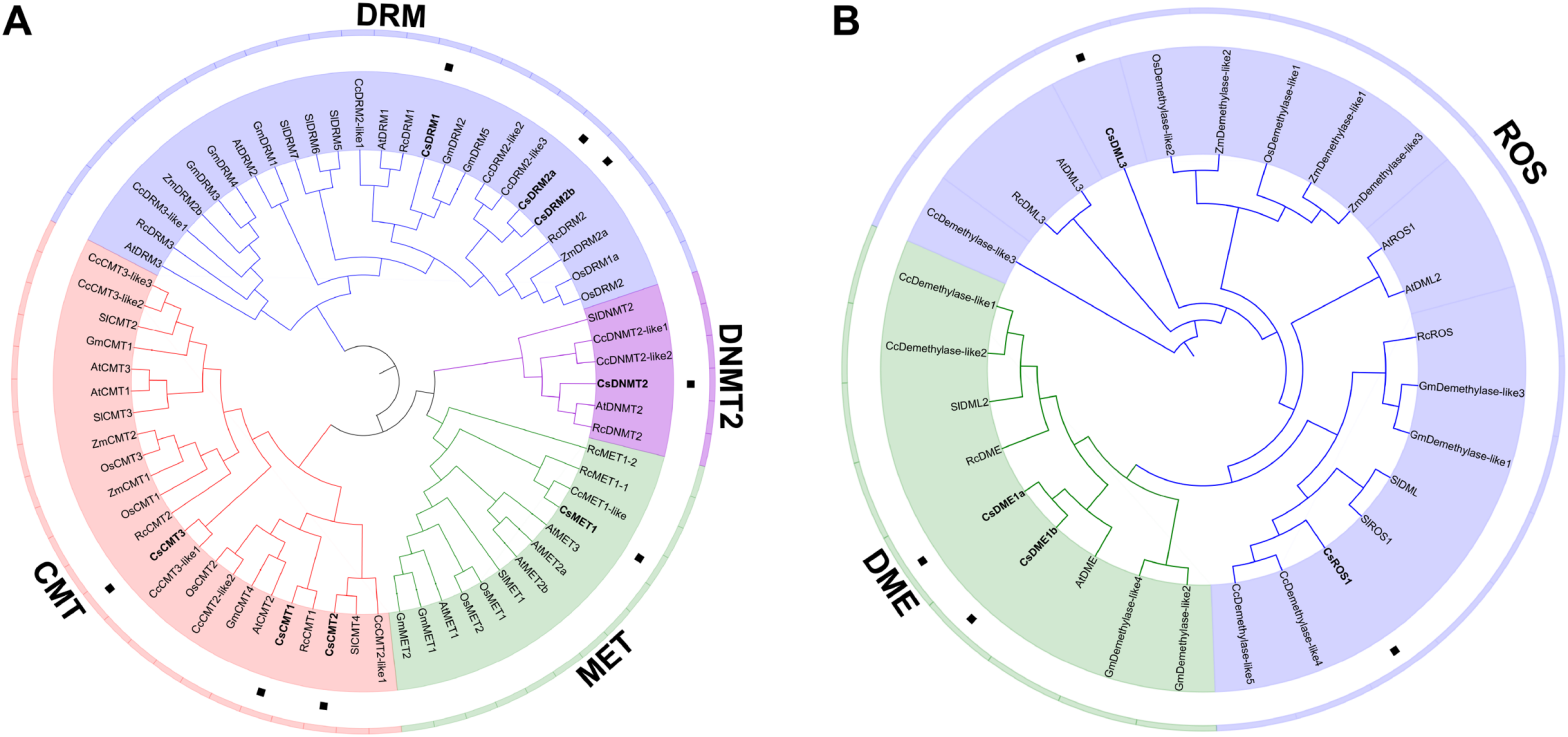
Phylogenetic analysis of the CsC5-MTases (A) and CsdMTases (B). Cs, *Camellia sinensis*; At, *Arabidopsis thaliana*; Rc, *Ricinus communis*; Cc, *Cynara cardunculus*; Sl, *Solanum lycopersicum*; Os, *Oryza sativa*; Zm, *Zea mays*; Gm, *Glycine max*.

To elucidate the phylogenetic relationship of C5-MTase and dMTase in plants, 67 C5-MTase protein sequences and 28 dMTase protein sequences from two monocotyledons (*O. sativa* and *Z. mays*) and six dicotyledons (*C. sinensis*, *A. thaliana*, *R. communis*, *C. cardunculus*, *S. lycopersicum*, and *G. max*) were used to construct phylogenetic trees (Table S3). As a result, the C5-MTase can be naturally grouped into four categories, namely, DRM, CMT, MET and DNMT2 (Fig. 3A). Compared to the DRM category, the MET, CMT and DNMT2 categories were more similar and belonged to the same clade. DNMT2 was the smallest category in the phylogenetic tree and contained only six members from five plant species. In the CMT and MET categories, most of the CMTs and METs in *O. sativa* and *Z. mays* were clustered into a small branch, respectively. Compared to monocotyledons in these two categories, most CMTs and METs in dicotyledons also form a distinct branch. In the largest DRM category, there is also a divergence between monocotyledons and dicotyledons. This result is consistent with the findings of previous studies on *S. lycopersicum* (Cao et al., 2014). It is speculated that C5-MTase may have different functions in monocotyledons and dicotyledons.

In phylogenetic classification, 28 dMTase proteins can be divided into two categories (Fig. 3B). Compared to the DME category, ROSs and DMLs showed a closer relationship. Therefore, CsROS1 and CsDML3 were grouped into ROS category, while CsDME1a and CsDME1b were classified into another DME category. Compared to ROS category, the nine proteins in the DME category were more dense. Similar to the classification of monocotyledons in C5-MTases, all dMTases in two monocotyledons (*O. sativa* and *Z. mays*) also form a distinct branch.

**Figure 4:**
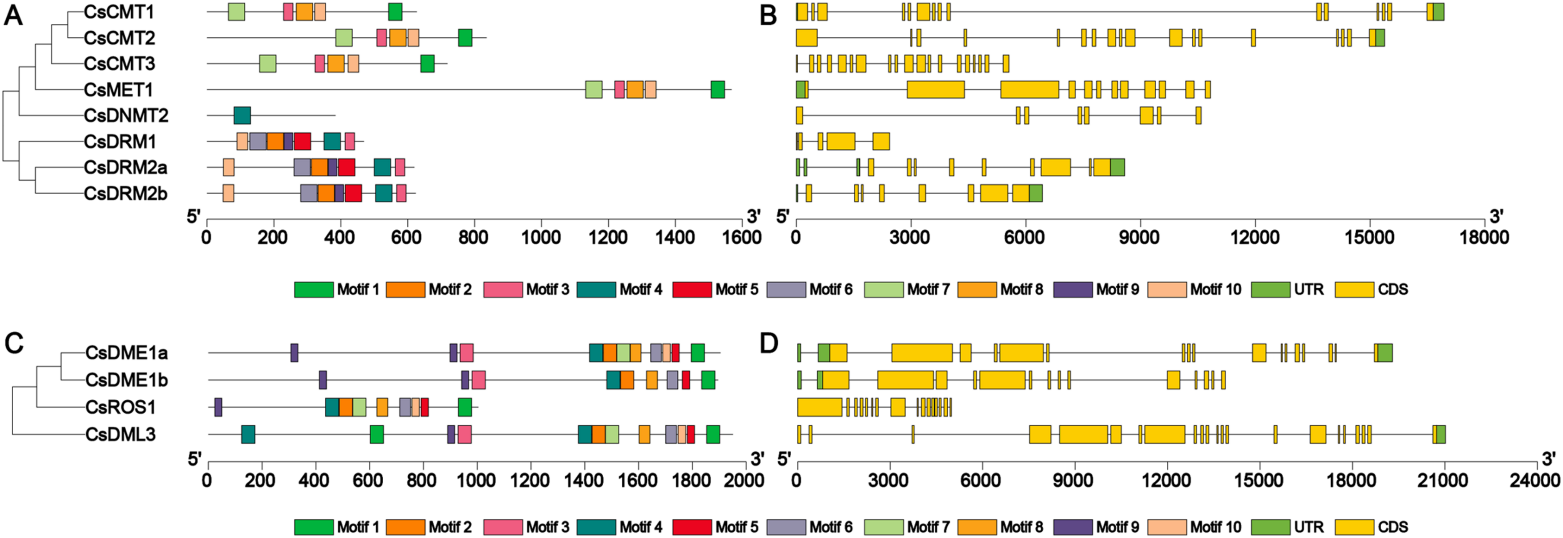
Phylogenetic relationships, conserved motifs, gene structures of CsC5-MTases and CsdMTases. (A) Phylogenetic relationships and conserved motifs of CsC5-MTases. (B) Gene structures of CsC5-MTases. (C) Phylogenetic relationships and conserved motifs of CsdMTases. (D) Gene structures of CsdMTases.

To further clarify the structural features of the *CsC5-MTase* and *CsdMTase* genes, the exon-intron structure of these genes was examined to elucidate the evolution of *CsC5-MTase* and *CsdMTase* in tea plants. The *CsC5-MTase* genes exhibited different exon-intron organizational patterns. In *CsC5-MTase* genes, *CsCMT* genes had the largest number of exons (Fig. 4). *CsCMT1*, *CsCMT2* and *CsCMT3* have 15, 18 and 19 exons, respectively. However, there are only five exons in *CsDRM1*. Moreover, the number of exons in the *CsdMTase* genes ranges from 15 to 22 (Fig. 4). Among them, the *CsDML3* gene contains 22 exons, which is the largest number of exons in *CsdMTase* genes. However, there are only 15 exons in *CsDME1b*. Further analysis revealed that all exon-intron junction sites of the *CsC5-MTase* and *CsdMTase* genes are GT-AG sites, which is consistent with the GT-AG splice rules in eukaryotes (Modrek & Lee, 2002).

Furthermore, the motifs in all members of CsC5-MTase and CsdMTase were analyzed. A total of 10 different motifs were identified in eight CsC5-MTase proteins (Table S4). Among the motifs, motifs 1, 3, 7, 8 and 10 were found in all CsCMTs and CsMET1. Hence, these two proteins were clustered together to be a small branch. The three CsDRMs were composed of 7 motifs, including motifs 3, 4, 5, 6, 8, 9 and 10. However, CsDNMT2 only contained motif 4, which has a large difference from the structure of CsDRMs. This finding may be why CsDNMT2 and CsDRMs belong to different branches. In the CsdMTase proteins, we also identified a total of 10 different motifs. Both CsDME1a and CsDME1b have all 10 motifs. In contrast, CsROS1 lacks motif 3, while CsDML3 has more than one motif 1 and 4. Therefore, both CsROS1 and CsDML3 differ from CsDMEs in their structure. To some extent, this finding is consistent with the results of the phylogenetic analysis. Consequently, CsC5-MTase and CsdMTase in the same cluster are similar in motif composition.

### Protein–protein interaction of CsC5-MTase and CsdMTase

**Figure 5:**
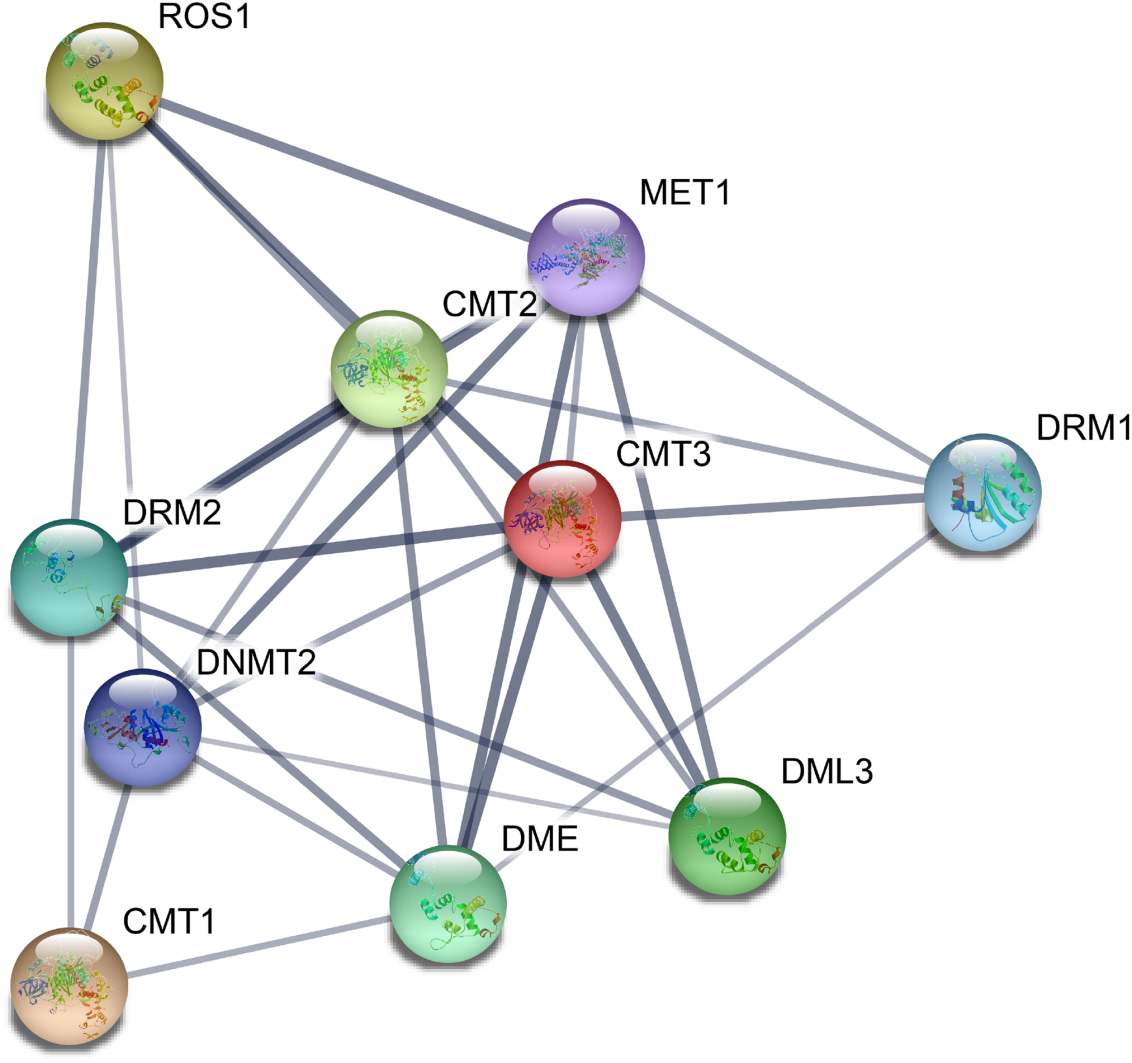
Potential protein–protein interaction network of CsC5-MTases and CsdMTases.

On the basis of the orthologs in *A. thaliana*, we constructed a protein–protein interaction network of CsC5-MTase and CsdMTase proteins using the STRING 11 tool. All eight CsC5-MTase and four CsdMTase are aligned to the corresponding AtC5-MTase and AtdMTase (Fig. 5). Among them, CsDRM2a and CsDRM2b were both homologous to AtDRM2. AtDME was the highest homologous protein of CsDME1a and CsDME1b. There were strong interactions among CMTs, METs and DRMs, and they may regulate the overall methylation level of plants by forming protein complexes. Moreover, we found that ROS1, DMEs and DMLs interact with three members of CMTs, indicating that plant methylation levels may be regulated by both C5-MTase and dMTase. Additionally, C5-MTase and dMTase may form a negative feedback loop that dynamically regulates the methylation level of plants.

### *Cis*-acting element analysis of *CsC5-MTase* and *CsdMTase* genes

**Figure 6:**
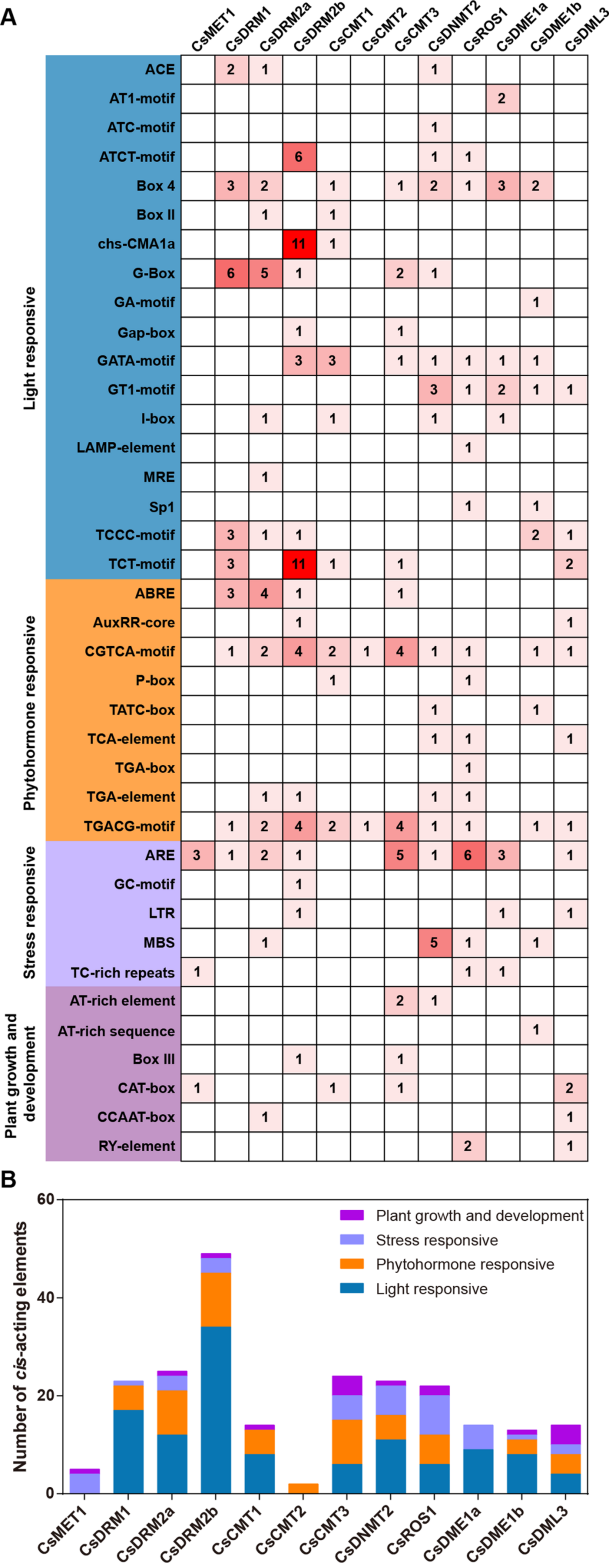
Analysis of the *cis*-acting elements in the promoters of *CsC5-MTases* and *CsdMTases*. (A) The numbers of different *cis*-acting regulatory elements in the promoters of *CsC5-MTases* and *CsdMTases*. (B) The *cis*-acting elements in four categories were represented by different colors.

To investigate the potential biological functions of CsC5-MTase and CsdMTase in tea plants, the upstream genome sequences (2,000 bp) of the start codon were analyzed using the PlantCARE tool. The identified *cis*-acting elements of CsC5-MTase and CsdMTase were divided into four categories, including light responsiveness, phytohormone responsiveness, stress responsiveness, and plant growth and development-related elements (Fig. 6). Among these elements, ARE, GC-motif, LTR, MBS and TC-rich repeat elements belong to the stress responsiveness classification. Except for *CsCMT1* and *CsCMT2*, the other 10 genes contain at least one *cis*-acting element involved in stress responsive classification, among which *CsROS1* has the largest number of stress-responsive elements. This finding suggested that the transcript abundance of these *CsC5-MTase* and *CsdMTase* genes may be affected by diverse forms of stresses. The light responsive elements are present in all *CsC5-MTase* and *CsdMTase* genes, except *CsMET1* and *CsCMT2*. In *CsMET1* and *CsDME1a*, we did not find any phytohormone response-related elements, but other *CsC5-MTase* and *CsdMTase* genes contain several phytohormone response-related elements, such as ABRE, AuxRR-core, and CGTCA-motif elements.

### Transcript abundance of *CsC5-MTase* and *CsdMTase* genes in tea plants under abiotic stress and withering processing

**Figure 7:**
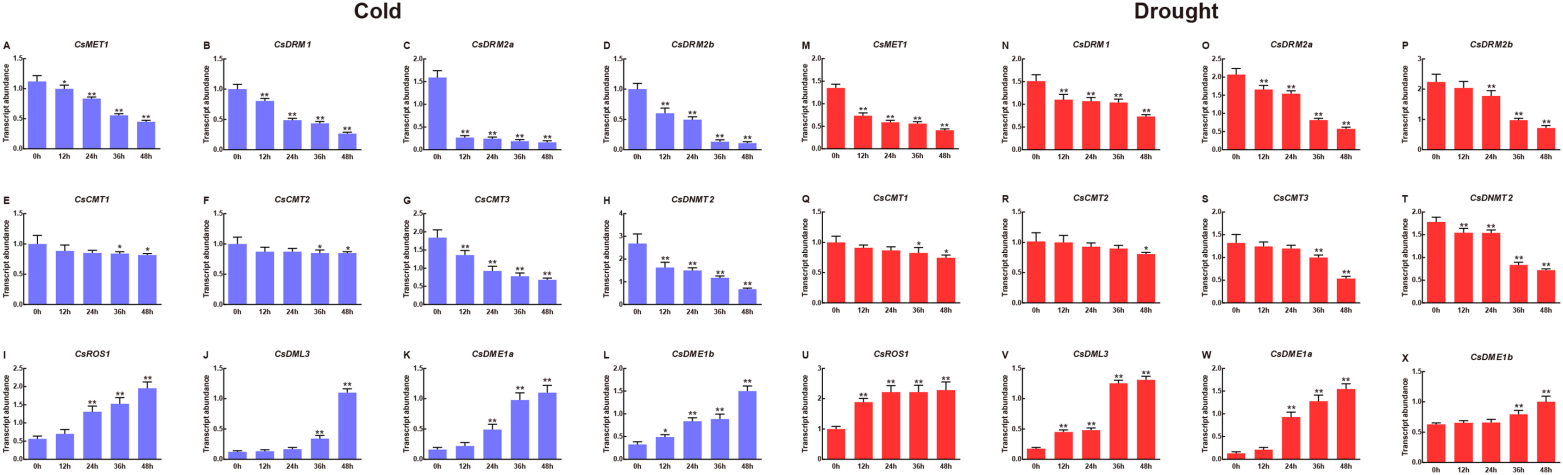
Transcript abundance of *CsC5-MTase* and *CsdMTase* genes under cold stress and drought stress. Transcript abundance of *CsMET1* (A), *CsDRM1* (B), *CsDRM2a* (C), *CsDRM2b* (D), *CsCMT1* (E), *CsCMT2* (F), *CsCMT3* (G), *CsDNMT2* (H), *CsROS1* (I), *CsDML3* (J), *CsDME1a* (K), and *CsDME1b* (L) under cold stress and *CsMET1* (M), *CsDRM1* (N), *CsDRM2a* (O), *CsDRM2b* (P), *CsCMT1* (Q), *CsCMT2* (R), *CsCMT3* (S), *CsDNMT2* (T), *CsROS1* (U), *CsDML3* (V), *CsDME1a* (W) and *CsDME1b* (X) under drought stress. ****Data are presented as mean ± standard deviation (SD). *indicates significant difference (*p* < 0.05) and **indicates highly significant difference (*p* < 0.01).

To clarify the potential roles of *CsC5-MTase* and *CsdMTase* genes involved in abiotic stress, we used qRT-PCR to determine the transcript abundance of *CsC5*-*MTase* and *CsdMTase* genes under cold and drought stresses. Under cold treatment, all members of the *CsC5*-*MTase* and *CsdMTase* genes showed significant changes in transcript abundance (Fig. 7). All eight *CsC5*-*MTase* genes were significantly reduced during cold treatment. At 12 h, the transcript abundance of these six genes (*CsMET1*, *CsDRM1*, *CsDRM2a*, *CsDRM2b*, *CsCMT3* and *CsDNMT2*) was significantly downregulated, whereas *CsCMT1* and *CsCMT2* genes showed no significant change in transcript abundance. With the extension of cold stress time, the transcript abundance of all *CsC5*-*MTase* genes showed a trend of continuous decline and reached the lowest point of transcript abundance at 48 h. Except for *CsCMT1* and *CsMET2*, the transcript abundance of six other *CsC5*-*MTase* genes was significantly suppressed at 48 h. In *CsdMTase* genes, the transcript abundance of all four genes was notably upregulated by cold treatment. The transcript abundance of *CsROS1* and *CsDME1a* was significantly induced at 24 h, while the transcript abundance of *CsDME1b* was significantly increased at 12 h. Then, the transcript abundance of all *CsdMTase* genes peaked at 48 h, which was significantly higher than that at 0 h. Hence, *CsC5-MTase* and *CsdMTase* genes are cold stress-specific response genes.

In response to drought stress, we observed that the transcript abundance of four *CsC5*-*MTase* genes (*CsMET1*, *CsDRM1*, *CsDRM2a* and *CsDNMT2*) changed immediately at 12 h, while the transcript abundance of the remaining *CsC5-MTase* genes was significantly inhibited at the late stage of drought stress. All the *CsC5-MTase* genes showed the lowest transcript abundance at 48 h. Compared with 0 h, the transcript abundance of the *CsCMT2* gene did not show a notably significant decline at 36 h, while the other seven *CsC5-MTase* genes reached a notably significant decline.

**Figure 8:**
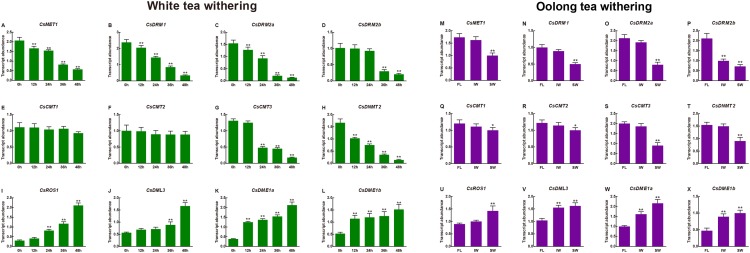
Transcript abundance of *CsC5-MTase* and *CsdMTase* genes under white tea withering and oolong tea withering. Transcript abundance ****of *CsMET1* (A), *CsDRM1* (B), *CsDRM2a* (C), *CsDRM2b* (D), *CsCMT1* (E), *CsCMT2* (F), *CsCMT3* (G), *CsDNMT2* (H), *CsROS1* (I), *CsDML3* (J), *CsDME1a* (K), and *CsDME1b* (L) under white tea withering and *CsMET1* (M), *CsDRM1* (N), *CsDRM2a* (O), *CsDRM2b* (P), *CsCMT1* (Q), *CsCMT2* (R), *CsCMT3* (S), *CsDNMT2* (T), *CsROS1* (U), *CsDML3* (V), *CsDME1a* (W) and *CsDME1b* (X) under oolong tea withering. FL, fresh leaves; IW, indoor-withered leaves; SW, solar-withered leaves. Data are presented as mean ± standard deviation (SD). *indicates significant difference (*p* < 0.05) and **indicates highly significant difference (*p* < 0.01).

Moreover, the transcript abundance of the *CsC5-MTase* and *CsdMTase* genes during withering processing of white tea and oolong tea was also investigated. As shown in Fig. 8, the transcript abundance of *CsC5-MTase* was significantly upregulated during withering processing of white tea. Among them, the transcript abundance of *CsMET1*, *CsDRM1*, *CsDRM2a*, *CsDRM2b*, *CsCMT3* and *CsDNMT2* was strongly repressed, whereas the transcript abundance of *CsCMT2* and *CsCMT3* was not altered significantly. In *CsdMTase* genes, the transcript abundance of four genes was significantly enhanced. At 12 h, the genes *CsDME1* and *CsDME2* were highly significantly upregulated, while *CsROS1* and *CsDML3* showed no significant change in transcript abundance. When white tea withered to 24 h and 36 h, the transcript abundance of *CsDML3* and *CsROS1* was also significantly induced. In general, the transcript abundance of all *CsC5-MTase* genes reached their lowest point at 48 h. In contrast, the *CsdMTase* genes had the highest transcript abundance at 48 h.

In different withering processing of oolong tea, the transcript abundance of all eight *CsC5-MTase* was downregulated at IW and SW compared to FL. In FL vs. IW, only the *CsDRM2b* transcript abundance was significantly inhibited, while the other seven *CsC5-MTase* were slightly changed in transcript abundance. The transcript abundance of *CsDML3*, *CsDME1a* and *CsDME1b* was dramatically induced and maintained at high levels in IW and SW. Although the transcript abundance of *CsROS1* was not affected in IW, the transcript abundance of this gene was significantly enhanced in SW. In SW, the transcript abundance of all *CsC5-MTase* genes was significantly downregulated, while the transcript abundance of four *CsdMTase* genes was significantly upregulated. In IW, only *CsDRM2b*, *CsDML3*, *CsDME1a* and *CsDME1b* showed significant change in transcript abundance.

## Discussion

With the release of tea reference genome, gene family identification, classification, and function prediction have gradually become new research hotspots (Chen et al., 2019; Liu et al., 2019; Ma et al., 2019; Wang et al., 2018). With the extreme global climate, low temperature stress and drought stress may adversely affect the growth and development of tea plants and the quality of tea products. Meanwhile, it has been widely reported that CsC5-MTase and CsdMTase modulate methylation levels to participate in various stress responses (Zhang, Lang & Zhu, 2018). Fresh leaves are also affected by various stresses during the withering process, including drought, heat, and UV/light radiation (Gui et al., 2015). Therefore, the study of *CsC5-MTase* and *CsdMTase* is of considerable significance for understanding the anti-stress mechanism and tea processing. The *C5-MTase* and *dMTase* genes have been well investigated in several plants (Cao et al., 2014; Gianoglio et al., 2017; Qian et al., 2014; Wang et al., 2016b). However, there is no report on the identification of these genes in tea plants. The functional roles of these genes in stress response and tea withering processing have not been determined. To elucidate DNA methylation regulation in *C. sinensis*, we identified eight *CsC5-MTase* and four *CsdMTase* genes based on the tea reference genome. The physical and chemical characteristics, phylogenetic relationships, gene structures, protein–protein interactions, and *cis-*acting elements of the genes were also investigated.

### Structural and evolutionary features of *CsC5-MTase* and *CsdMTase* in tea plant

Compared with other plant species, a total of eight *C5-MTase* and four *dMTase* genes have been identified in *C. sinensis*. However, the number of these two gene families discovered in *A. thaliana* (15), *C*. *cardunculus* (17) and *G. max* (13) is higher than that of tea plants (12). Due to the tea plant genome (3.02 GB) being approximately several-fold larger than that of *A. thaliana* (125 MB) (Cheng et al., 2017), *R. communis* (350 MB) (Chan et al., 2010) and *G. max* (978 MB) (Schmutz et al., 2010), indicating that *CsC5-MTase* and *CsdMTase* may have lost events in tea plants. Moreover, previous research has confirmed several gene contraction and expansion events occurred during the evolution of tea plants (Xia et al., 2017). Several studies have suggested that the contraction and expansion of these genes will directly affect the gene number in various plant species (Bennetzen, 2002; Choi, Jansen & Ruhlman, 2019; Lehtonen & Cárdenas, 2019). Hence, we inferred that gene duplication and loss events may be the key factors in the evolution of these two gene families. This finding is consistent with previous studies in maize (Qian et al., 2014) and globe artichoke (Gianoglio et al., 2017).

In phylogenetic classification, *C5-MTase* and *dMTase* genes in monocotyledons (*O. sativa* and *Z. mays*) are clustered in different branches from those in dicotyledons. It is speculated that the functions of *C5-MTase* and *dMTase* may be different between monocotyledons and dicotyledons. Consistent with previous research (Cao et al., 2014; Gianoglio et al., 2017), dicotyledons and monocotyledons may adopt different strategies in the evolution of *C5-MTase* and *dMTase*. Based on C5-MTase domain conservation, CsC5-MTase is classified into four distinct subfamilies, namely, MET, CMT, DRM and DNMT2. This classification is consistent with other dicotyledons, except soybean (lack of DNMT2). Similar to the *MET* gene in *S*. *lycopersicum* and *C*. *cardunculus*, only one *MET* gene was identified in tea plants, while in other plant species (*A. thaliana*, *R. communis*, *O. sativa*, *G. max* and *Z. mays*), each contains more than two *MET* genes. The number of *CMT* genes varies greatly among different plant species. In tea plants, a total of three *CMT* genes were identified. Similarly, three *CMT* genes were found in *A. thaliana*, *S*. *lycopersicum* and *O. sativa*, respectively. However, a total of four *CMT* genes were discovered in *C*. *cardunculus*. Compared to *A. thaliana* and *R. communis*, no *DRM3* was found in the tea genome, suggesting that the *DRM3* gene may be lost during the evolution of tea plants. At the same time, this effect may also be related to the presence of two *CsDRM2* genes in the tea genome. Consistent with a previous study (Blanc & Wolfe, 2004), the function of *DRM3* may be replaced by *DRM2a* and *DRM2b*. In contrast to the absence of *DNMT2* in *O. sativa* and *G. max*, the tea plant genome harbors one *CsDNMT2* gene. Previous studies have shown that DNMT2 has a wide substrate specificity, not only as a DNA methylase (Ashapkin, Kutueva & Vanyushin, 2016) but also as an RNA methylase to participate in various pathways to regulate the methylation level (Hermann, Schmitt & Jeltsch, 2003; Tuorto et al., 2012). Hence, the presence of CsDNMT2 may diversify the ways in which methylation levels are regulated in response to various stresses. In all plant species analyzed to date, the number of *dMTase* genes is considerably smaller than that in *C5-MTase*. In the present study, the number of *dMTase* genes in different plants ranged from 3 to 5. There is little difference in the number of these genes among different plant species. Based on the analysis of phylogenetic classification, *dMTase* genes can be divided into three distinct subfamilies, including *ROS*, *DML* and *DME*. In tea plants, all three subfamilies of *dMTase* genes were identified. Among the subfamilies, *CsDME* has two highly similar members, namely, *CsDME1a* and *CsDME1b*. These genes are closer in the tea genome, suggesting that they may result from localized replication events. Recent research also confirmed that methylation-related genes are affected by genome duplication and tandem replication (Pei et al., 2019). In total, the loss of *CsDRM3* and duplication of *CsDME* suggested that *CsC5-MTase* and *dMTase* genes have functional redundancy and divergence.

Although the number of *C5-MTase* and *dMTase* genes varies widely among different plant species, the conserved domains contained in these proteins are similar. In this study, all members of CsC5-MTase contained one DNA-methylase domain, indicating that this is the core conserved domain of CsC5-MTase. The absence of this domain will make C5-MTase proteins unable to normally methylate the C-5 of cytosine in DNA (Wang, Lou & Li, 2019; Zhang, Lang & Zhu, 2018). In CsC5-MTase, the three members of CsDRM and CsDNMT2 only contain the DNA-methylase domain, while another domain (chromo domain) exists in CsCMT. The chromo domain is involved in chromatin interactions and is thought to mediate recognition and binding to target DNA. It was also reported that the chromo domain is responsible for gene regulation associated with chromatin remodeling, which can also bind to methylated histones, thereby affecting the methylation level of the genome (Jones, Cowell & Singh, 2000; Tajul-Arifin et al., 2003). Unlike CsCMT1, both CsCMT2 and CsCMT3 have a BAH domain. The BAH domain is involved in gene silencing and replication by mediating the association between chromatin and heterochromatin regions and protein–protein interaction (Saze et al., 2013). Combined with previous studies, the maintenance of CHG methylation in plants is catalyzed by CMT2 and CMT3 (Lindroth et al., 2001; Stroud et al., 2014). Similarly, it has been reported that the BAH domain in AtCMT1 is disrupted by frameshift mutation; therefore, AtCMT1 cannot function normally in methylation maintenance (Bewick et al., 2017). Hence, we inferred that the BAH domain is the key conserved structural domain for the methylation maintenance of CMT. However, CsCMT1 without this structural domain may have a limited contribution to affecting methylation level in tea plants, while CsCMT2 and CsCMT3 may be the key factors regulating the methylation level of tea plants. Additionally, we performed conserved domain identification of CsMET1. A total of five conserved domains were identified in CsMET1, including two DNMT1-RFD domains, two BAH domains, and one DNA-methylase domain. Compared to CsCMT2 and CsCMT3, CsMET1 has two extra DNMT1-RFD domains, which are responsible for the recognition of hemi-methylated CG dinucleotides and the methylation of unmodified cytosine (He, Chen & Zhu, 2011). We speculate that the presence of the DNMT1-RFD domain is an essential element for CsMET1 to participate in the maintenance of CG methylation.

Demethylase is an important factor involved in the dynamic regulation of methylation (Kapoor, Agius & Zhu, 2005). To further understand the specific functions of these genes in tea plants, the recognizable domains in all CsdMTases were also analyzed. All four CsdMTases contain an RRM-DME domain at their C-terminus. The RRM-DME domain is responsible for catalyzing the removal of 5mC base, which can effectively reduce the methylation level of plants (Iyer, Abhiman & Aravind, 2011). Moreover, CsROS1 and CsDML3 contain one Perm-CXXC domain, which is not found in two members of CsDMEs. This domain catalyzes the release of 5mC in DNA by a glycosylase/lyase mechanism, thereby effectively reducing the methylation level of plants (Mok et al., 2010; Morales-Ruiz et al., 2006). Genes with the same domain may have similar functions; therefore, CsROS1 and CsDML3 may play similar roles in plant demethylation. which is consistent with the result that these two genes belong to the same branch in the phylogenetic classification.

In total, we suggested that there is a difference in the number of *CsC5-MTase* and *CsdMTase* genes compared to monocotyledons, which may be related to gene replication and gene loss events in tea plant. Combined with loss of *CsDRM3* and duplication of *CsDME* in tea plants, gene replication and loss events enrich the functional redundancy and divergence of C5-MTase and dMTase. Meanwhile, the number of *CsC5-MTase* and *CsdMTase* genes is not highly different from those of other dicotyledonous plants. Similar to other dicotyledons, the gain and loss of *CsC5-MTase* and *CsdMTase* genes experienced relaxed natural selection. In the evolutionary process of *C5-MTase* and *dMTase*, gene duplication and loss events promoted the abundant functions of *CsC5-MTase* and *CsdMTase* such that these genes have different roles in the regulation of methylation level of tea plant. This finding is consistent with the results of previous research (Gu et al., 2016; Wang et al., 2016b).

### *CsC5-MTase* and *CsdMTase* genes play crucial roles in response to abiotic stress in tea plants and withering processing

In the production of tea plants, cold and drought are the major abiotic stresses that are not conducive to the growth and development of tea plants. Recent studies have shown that DNA methylation plays an important role in the plant stress response (Sanchez & Paszkowski, 2014; Xu et al., 2015). However, the transcript abundance of *CsC5-MTase* and *CsdMTase* genes in tea plants under these two abiotic stresses has rarely been studied. In this present study, we investigated the transcript abundance of the *CsC5-MTase* and *CsdMTase* genes under cold and drought stresses. These results showed that the transcript abundance of all *CsC5-MTase* and *CsdMTase* genes revealed dynamic trends at certain times from 0 to 48 h under cold and drought stresses, demonstrating a possible regulatory role of DNA methylation in the abiotic stress of tea plants. Except for *CsCMT1* and *CsCMT2*, the transcript abundance of the other six *CsC5-MTase* genes showed a significant decrease after 12 h of cold treatment. With the extension of cold stress time, the transcript abundance of all *CsC5*-*MTase* genes showed a trend of continuous decline and reached the lowest point of transcript abundance at 48 h. In contrast, the transcript abundance of all *CsdMTase* genes showed a gradual increase under cold stress, reaching their highest transcript abundance at 48 h. In drought stress, the transcript abundance of *CsC5-MTase* and *CsdMTase* genes was similar under cold stress, and all *CsC5-MTase* genes also showed a descending trend under drought stress, while the transcript abundance of all *CsdMTase* genes increased significantly with increasing drought stress time. The results are consistent with the response of *C5-MTase* and *dMTase* to abiotic stress in *R*. *communis* (Victoria et al., 2018). These results indicated that these two gene families played different roles in the abiotic stress response.

Increasing evidences have shown that withering plays a predominant role in affecting the quality of white tea and oolong tea (Hu et al., 2018; Ma et al., 2018; Wang et al., 2019b). Similar to the effects of stress on tea plants, FL are also affected by various stresses during withering processing, including drought, high temperatures and UV/light radiation. To understand the role of the *C5-MTase and dMTase* genes in the withering process, the transcript abundance of these two gene families during the withering processing of white tea and oolong tea was analyzed. During withering of white tea, the transcript abundance of six *C5-MTase* genes (*CsMET1*, *CsDRM1*, *CsDRM2a*, *CsDRM2b*, *CsCMT3* and *CsDNMT2*) was significantly suppressed with the extension of withering time. However, the transcript abundance of two *CsCMT* genes (*CsCMT1* and *CsCMT2*) was only slightly (not significantly) downregulated. On the other hand, all four *CsdMTase* genes were significantly induced, and their transcript abundance was sharply increased. This indicated that the effects of withering on white tea might result from the transcriptional regulation of methylation-related genes.

Among oolong tea withering, solar-withering and indoor-withering are the most common processing methods (Lin et al., 2013). The flavor of tea products after different processing is quite different. In the withering of oolong tea, withered leaves will suffer from diverse types of stresses, such as drought, heat, and UV/light radiation. Combined with the above results, *CsC5-MTase* and *CsdMTase* may play different roles in solar-withering and indoor-withering. To understand the specific functions of *CsC5-MTase* and *CsdMTase* genes in solar-withering and indoor-withering, we investigated the transcript abundance of *CsC5-MTase* and *CsdMTase* genes in FL, IW and SW, respectively. In this study, we found that the transcript abundance of all *CsC5-MTase* showed a decrease in both IW and SW compared to FL. However, except for *CsDRM2b*, the transcript abundance of the other seven *CsC5-MTase* genes showed only a slight (not significant) decrease in IW compared to FL. In FL vs. SW, the transcript abundance of all *CsC5-MTase* was significantly inhibited. Compared with IW, the transcript abundance of these *CsC5-MTase* genes in SW was also significantly reduced. In these three samples, all eight *CsC5-MTase* showed lowest transcript abundance in SW. In the *CsdMTase* genes, the transcript abundance of these four genes is opposite to that of *CsC5-MTase*. All four *CsC5-MTase* were present at the highest transcript abundance in SW. Specifically, *CsROS1* showed no significant change in transcript abundance between FL and IW, while the transcript abundance of *CsDML3*, *CsDME1a* and *CsDME1b* in IW was significantly higher than those in FL. Consistent with the detection of the transcript abundance of *CsC5-MTase* and *CsdMTase* genes during abiotic stress and white tea withering, the transcript abundance of six *C5-MTase* genes (*CsMET1*, *CsDRM1*, *CsDRM2a*, *CsDRM2b*, *CsCMT3* and *CsDNMT2*) was significantly suppressed in SW, while the transcript abundance of all four *CsdMTase* genes was significantly induced. In the withering of oolong tea, the SW will suffer from different types of stress, including drought, heat, and UV/light radiation, while the indoor withered leaves are primarily affected by drought stress. Hence, solar-withering accelerates the dehydration process of tea leaves, and the stress degree in SW was deeper than that in IW. Due to the combination of multiple stresses, SW suffers a considerably higher stress degree than IW. Based on the above results, we concluded that the transcript abundance of *CsC5-MTase* and *CsdMTase* genes may be related to the stress degree of withered leaves. Similarly, previous work has demonstrated that C5-MTase and dMTase play important roles in response to salt stress (Liu et al., 2018b), cold stress (Gu et al., 2016), and drought stress (Victoria et al., 2018). Moreover, we observed a large number of light-responsive and stress-responsive *cis*-acting elements in the promoter regions of *CsC5-MTase* and *CsdMTase*. It has been reported that *cis*-acting elements are important molecular switches involved in dynamic network transcriptional regulation of gene activity and affect the transcript abundance of relevant genes (Shinozaki, Yamaguchi-Shinozaki & Seki, 2003; Yamaguchi-Shinozaki & Shinozaki, 2005). Consistent with a previous study (Cheng et al., 2013), these *cis*-acting elements have stress-related functions in *CsC5-MTase* and *CsdMTase*, which could respond to diverse stress signals and affect the transcript abundance of *CsC5-MTase* and *CsdMTase*. This effect may explain the significant changes in the transcript abundance of these two gene families under abiotic stress and withering processing. The presence of abundant light-responsive elements explained that the transcript abundance of *CsC5-MTase* and *CsdMTase* is affected by solar light. Compared with FL, the change of most *CsC5-MTase* and *CsdMTase* in SW are greater than that of IW. Of note is the observation that we did not identify a *cis*-acting element associated with stress-responsive in the promoter regions of the *CsCMT1* and *CsCMT2* genes. This may be the reason why the difference in transcript abundance between *CsCMT1* and *CsCMT2* genes is less than that in other *CsC5-MTase* during stress and withering.

**Figure 9:**
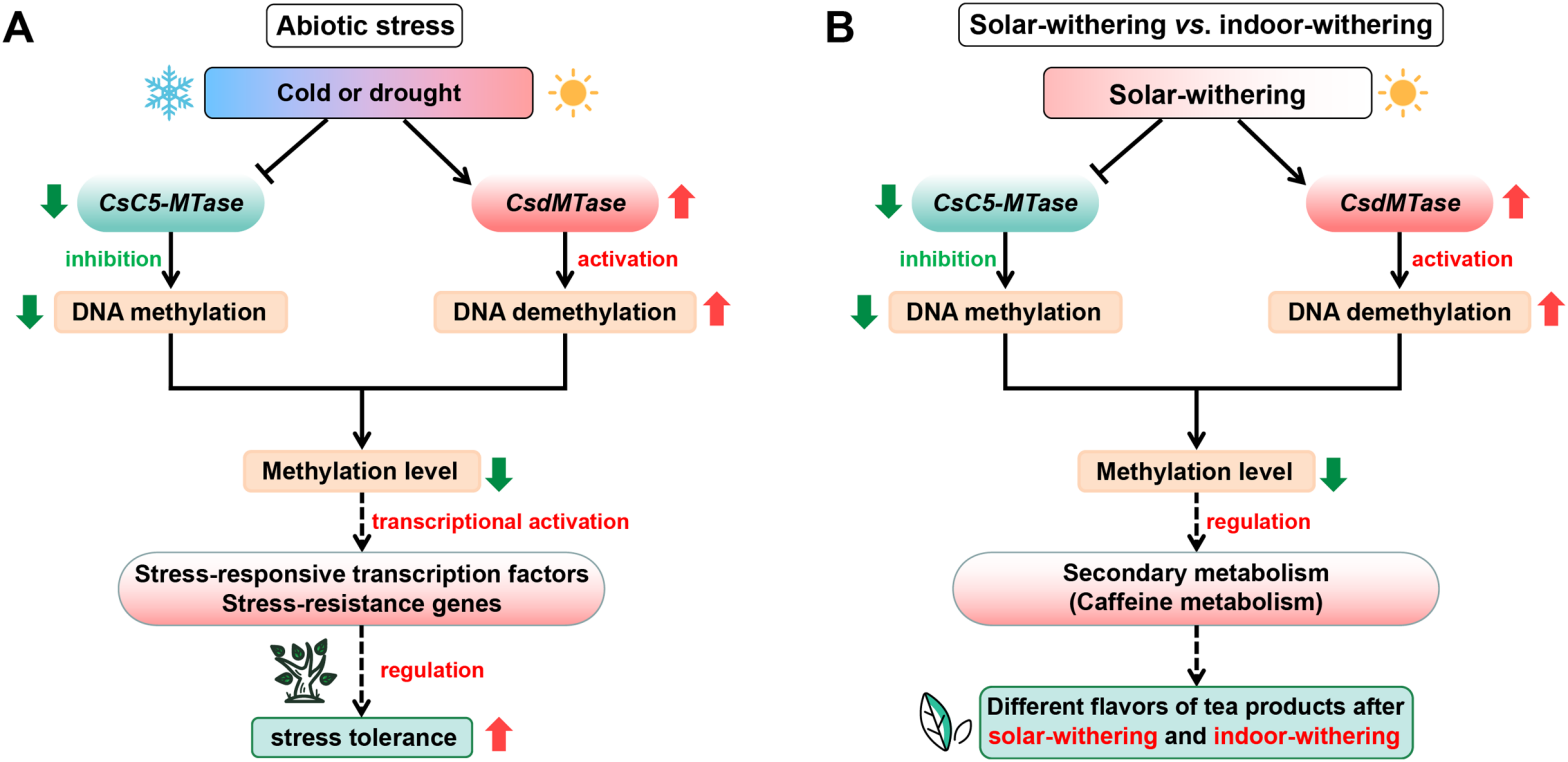
Schematic illustration showing the roles of *CsC5-MTase* and *CsdMTase* under abiotic stress (A) and withering treatment (B). The solid line with the arrow represents direct regulation; the dashed line with the arrow represents indirect regulation; the T-shaped line represents inhibition.

The transcript abundance of *CsC5-MTase* and *CsdMTase* directly affect the dynamic change of plant methylation levels (Zemach et al., 2010). Combined with the above results, this suggested that the DNA methylation patterns of tea plants might undergo a dramatic change during abiotic stress and withering. With the upregulation of *CsdMTase*, the DNA demethylation mechanism in tea plants is activated. Recent studies also revealed that the level of plant methylation is significantly reduced in response to abiotic stress (Dhar et al., 2014; Furner & Matzke, 2011). Moreover, hypomethylation level in DNA methylase mutants enhances resistance to biotrophic pathogens (López Sánchez et al., 2016). Under normal conditions, the promoter region of most genes in plants is maintained at a high methylation level. When plants are considered to be under stress, methylation markers from regulatory regions of stress-responsive transcription factors and other stress-resistance genes will be erased by demethylation mechanisms, which may enhance the ability of tea plants to cope with various stresses. In accordance with the studies in *Hevea brasiliensis* (Uthup et al., 2011) and *R. communis* (Victoria et al., 2018), the reduction of DNA methylation level may facilitate the activation of transposon transcription, and transposon transcription is often accompanied by stress conditions, thereby further activating the transcript abundance of related stress-resistant genes. Moreover, it has been reported that DNA methylation is involved in the regulation of secondary metabolism (Bharti et al., 2015; Conde et al., 2017). Caffeine is one of the most important secondary metabolites and plays an important role in tea flavor. In the caffeine metabolism, several METs participate in caffeine metabolism and convert xanthine to caffeine, and the methyl donor is also derived from S-adenosyl methionine (Deng et al., 2008; Kato et al., 1996). Meanwhile, S-adenosyl methionine is a methyl donor for cytosine methylation. Hence, methylation levels may be closely related to caffeine metabolism in tea plants. Increasing evidence has shown that drought stress and withering treatment can significantly affect the caffeine content in tea plants (Wang et al., 2016c, 2019b; Ye et al., 2018). Combined with the dynamic transcript abundance of *CsC5-MTase* and *CsdMTase* during abiotic stress and withering processing, it is suggested that *CsC5-MTase* and *CsdMTase* could affect caffeine metabolism in tea plants by regulating the methylation level. Due to the different transcript abundance of *CsC5-MTase* and *CsdMTase* in IW and SW, this may be one of the reasons for the different flavors of tea products after indoor-withering and solar-withering. On the basis of our data, we propose a hypothetical model to explain potential functions of *CsC5-MTase* and *CsdMTase* in abiotic stress and different withering processing (Fig. 9).

## Conclusions

Based on the tea reference genome, a total of eight *CsC5-MTase* and four *CsdMTase* genes were identified. Phylogenetic classification revealed that *CsC5-MTase* genes were divided into four subfamilies, including *CsMET*, *CsCMT*, *CsDRM* and *CsDNMT2*. These *CsdMTase* genes can be classified into *CsROS*, *CsDME* and *CsDML* subfamilies. The conserved domain analysis of these two gene families showed that gene loss and duplication events may occur in the evolution of *CsC5-MTase* and *CsdMTase*. Analysis of *cis*-acting elements revealed that these genes may be involved in a wide range of biological processes, including stress response, phytohormone response, and plant growth and development. In addition, the transcript abundance of *CsC5-MTase* and *CsdMTase* genes under abiotic stress (cold and drought) and withering processing (white tea and oolong tea) were detected, and the results showed that *CsC5-MTase* and *CsdMTase* genes may play vital roles in regulating responses to abiotic stress. Furthermore, these *CsC5-MTase* and *CsdMTase* may affect tea flavor in withering processing. We hope that the study helps to elucidate the possible roles of *CsC5-MTase* and *CsdMTase* in the abiotic stress response of tea plants and in the flavor of tea in withering processing and also provides a foundation for further understanding the epigenetic regulatory mechanism in tea plants.

## Supplemental Information

10.7717/peerj.8432/supp-1 Supplemental Information 1 Basic information of C5-MTases and dMTases from *A. thaliana* and *S. lycopersicum*.

10.7717/peerj.8432/supp-2 Supplemental Information 2 Primers used for qRT-PCR analyses.

10.7717/peerj.8432/supp-3 Supplemental Information 3 C5-MTases and dMTases used for the construction of phylogenetic trees.

10.7717/peerj.8432/supp-4 Supplemental Information 4 Consensus sequences of motifs identified in CsC5-MTases and CsdMTases in tea plant.

## Additional Information and Declarations

### Data Availability

The following information was supplied regarding data availability: The raw data are available in Tables S1–S4.
